# Supplementary material for: Genetic and microenvironmental intra-tumor heterogeneity impacts colorectal cancer evolution and metastatic development
Source: Commun Biol. 2022 Sep 9;5:937. doi: 10.1038/s42003-022-03884-x (PMC9463147; doi:10.1038/s42003-022-03884-x)
Supplement: Supplementary file 2 — Supplementary Information [file 42003_2022_3884_MOESM2_ESM.pdf]

Supplementary Table 1

	Our Cohort
No. of patients	112
**Gender**	
Male	44 (39%)
Female	68 (61%)
Median age (range)	70 (36-91)
**Stage**	
II	65 (58%)
III	47 (42%)
**Location**	
left	33 (30%)
right	68 (61%)
rectum	5 (4%)
other/unclassified	6 (5%)
**Relapse**	
Non-Metastatic	92 (82%)
Metastatic	20 (18%)
Median time to relapse [years] (range)	1.0 (0.21-2.9)
**Overall survival**	
Deceased	28 (25%)
Median time to death [years] (range)	1.8 (0.5-5.6)
Alive	84 (75%)
Median follow up time [years] (range)	3.1 (0-11.5)

Supplementary Table 1. Clinico-pathologic features of 112 early-stage CRC samples.

Supplementary Figure 1

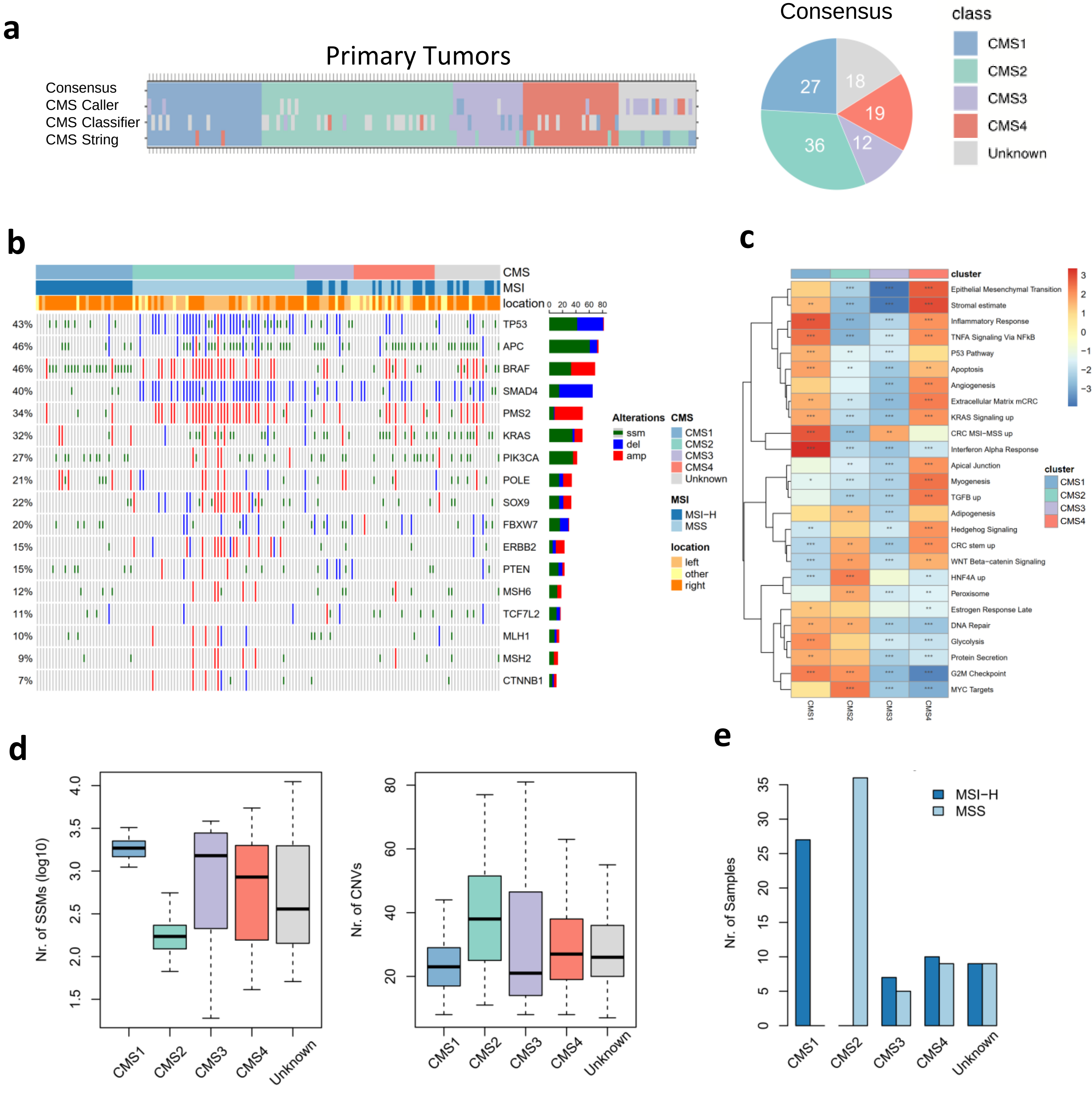

Supplementary Figure 1. Molecular and clinical features of CMS. a) Consensus classification of 112 CRC early stage (II/III) based on diferent tools: CMS caller; CMS Classifier and CMS String. **b)** Oncoprint of CRC early-stage CRC samples depicting simple somatic mutations (SSMs) and copy number variants (CNVs) divided in amplifications and deletions. **c)** Gene set enrichment analysis of CMS signatures from CMScaller R package. Colors correspond to normalized enrichment score (NES) values, comparing the expression of genes for each category in samples of one CMS against samples of the other CMSs (adjusted p-value *: < 0.05; **: <0.01; ***: <0.001) **d)** Distribution of simple somatic mutatios (SSMs), copy number variants (CNVs) for each CMS group. **e)** Number of samples with high Microsatellite instability (MSI-H) and Microsatelite Stability (MSS).

Supplementary Figure 2

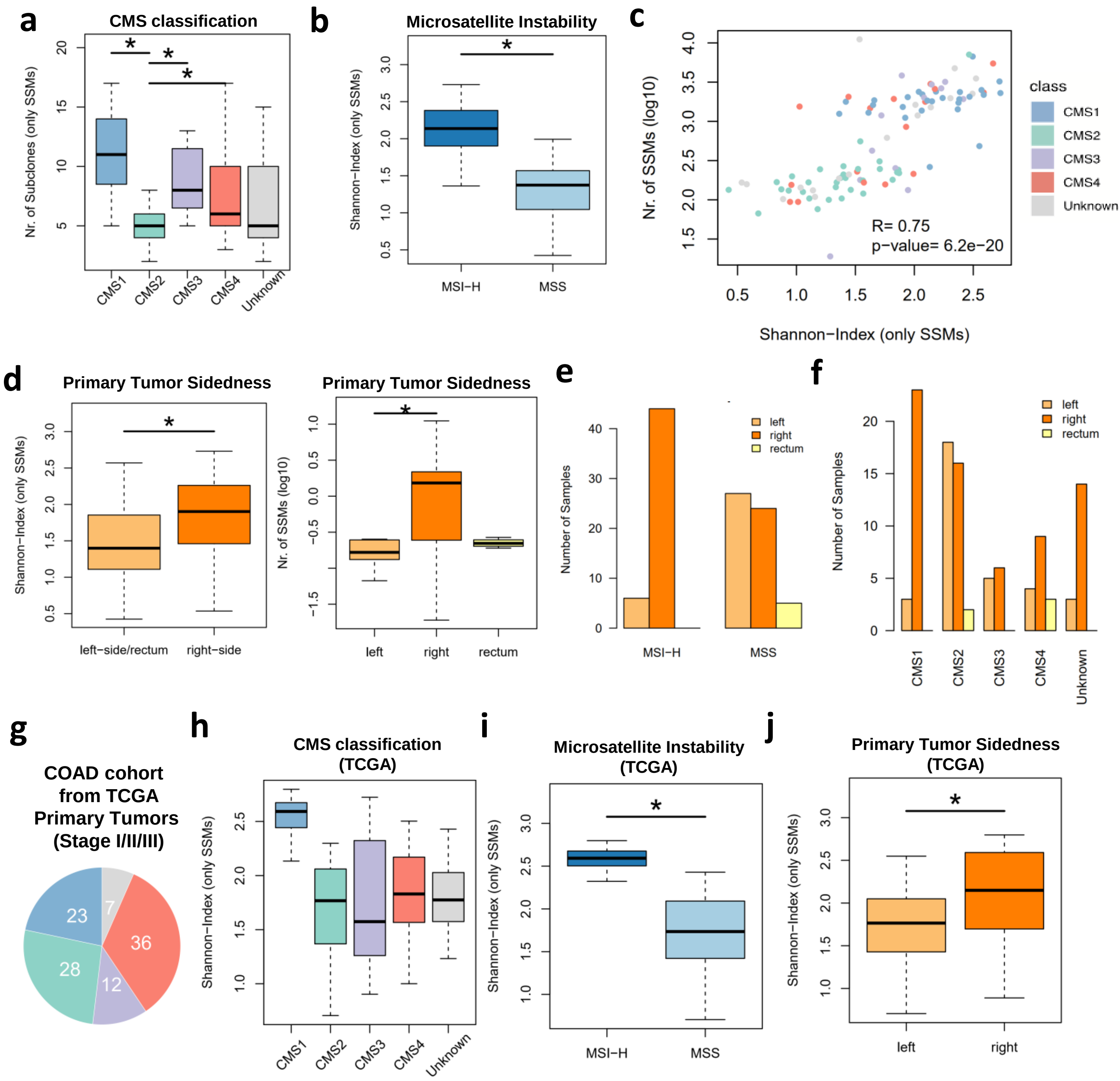

Supplementary Figure 2. Genetic Inter- and Intra-Tumor Heterogeneity of CRC using Expands (only SSMs). a) Distribution of the number of subclones based on Expands clonal composition segregated by CMS subtype; **b)** Distribution of the Shannon-Index values based on Expands clonal composition segregated by Microsatellite Instability Status. **c)** Comparison between ITH levels (only SSMs) and number of SSMs. Colors indicate CMS subtype for each CRC sample. Estimate and statistical significance of the Pearson correlation are presented; **d)** Distribution of of the Shannon-Index values based on Expands clonal composition and number of SSMs (log10) for left, right-sided and rectal tumors. **e-f**) Primary tumor location of CRC samples segregated according to MSI status (**e**) and CMS groups (**f**). **g**) Consensus classification of 106 CRC early stage from TCGA. **h-j**) Distribution of the Shannon-Index values based on Expands clonal composition of CRC TCGA samples segregated by CMS subtype (**h**); Microsatellite Instability Status (**i**) or primary tumor location (**j**). **Wilcoxon signed-rank test p-value < 0.05.

Supplementary Figure 3

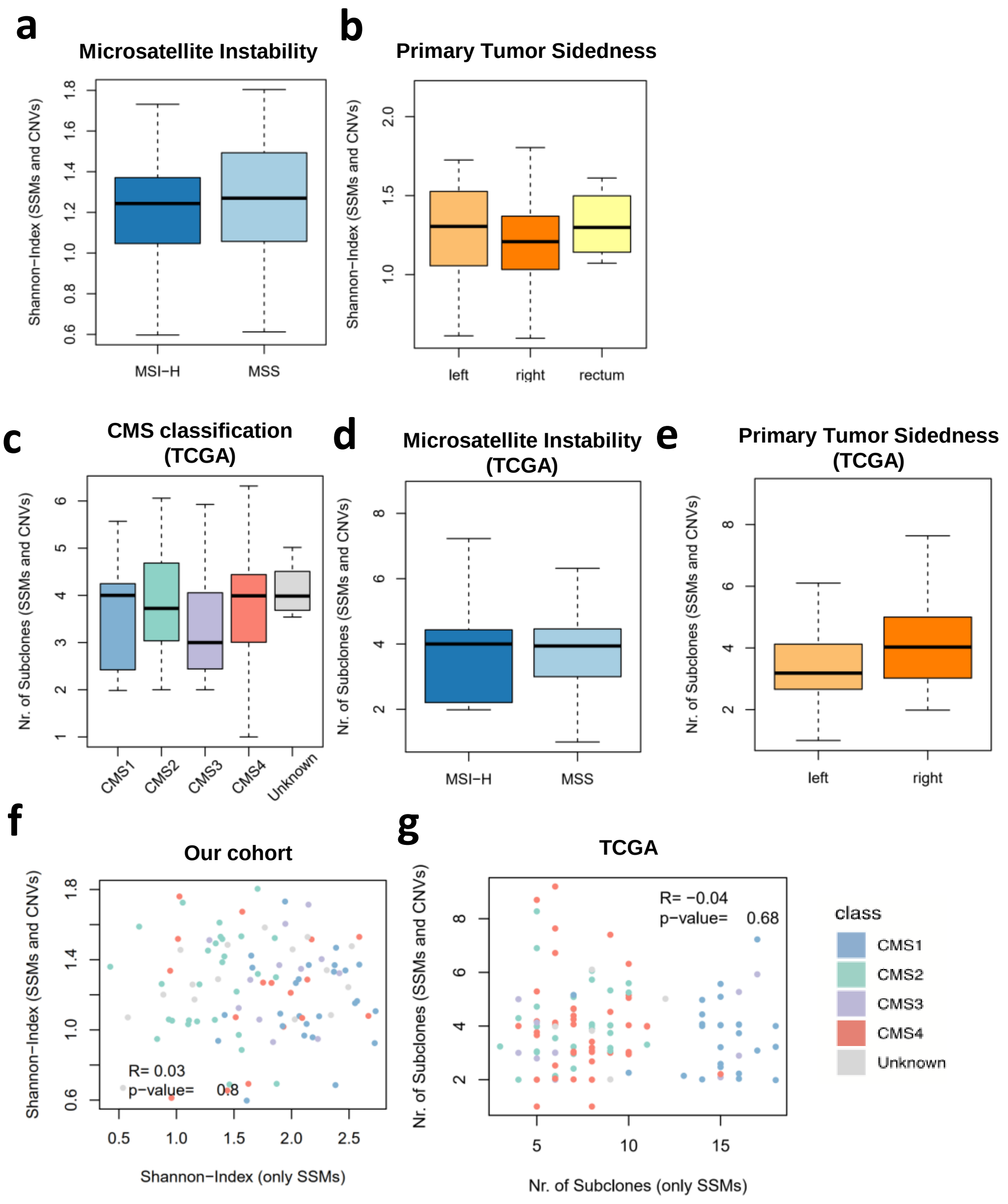

Supplementary Figure 3. Genetic Inter- and Intra-Tumor Heterogeneity of CRC using PhyloWGS (based on SSMs and CNVs). a-b) Distribution of the Shannon-Index values based on PhyloWGS clonal composition segregated by Microsatellite Instability status (**a**) and primary tumor location (**b**); **c-e**) Distribution of the number of subclones based on PhyloWGS for CRC TCGA samples segregated by CMS subtype (**c**); Microsatellite Instability Status (**d**) or primary tumor location (**e**). **f-g**) Comparison between both measures of ITH levels assessed solely using only SSMs or combineing both SSMs and CNVs in our cohort (**f**) and TCGA cohort (**g**). and number of SSMs. Colors indicate CMS subtype for each CRC sample. Estimate and statistical significance of the Pearson correlation are presented.

Supplementary Figure 4

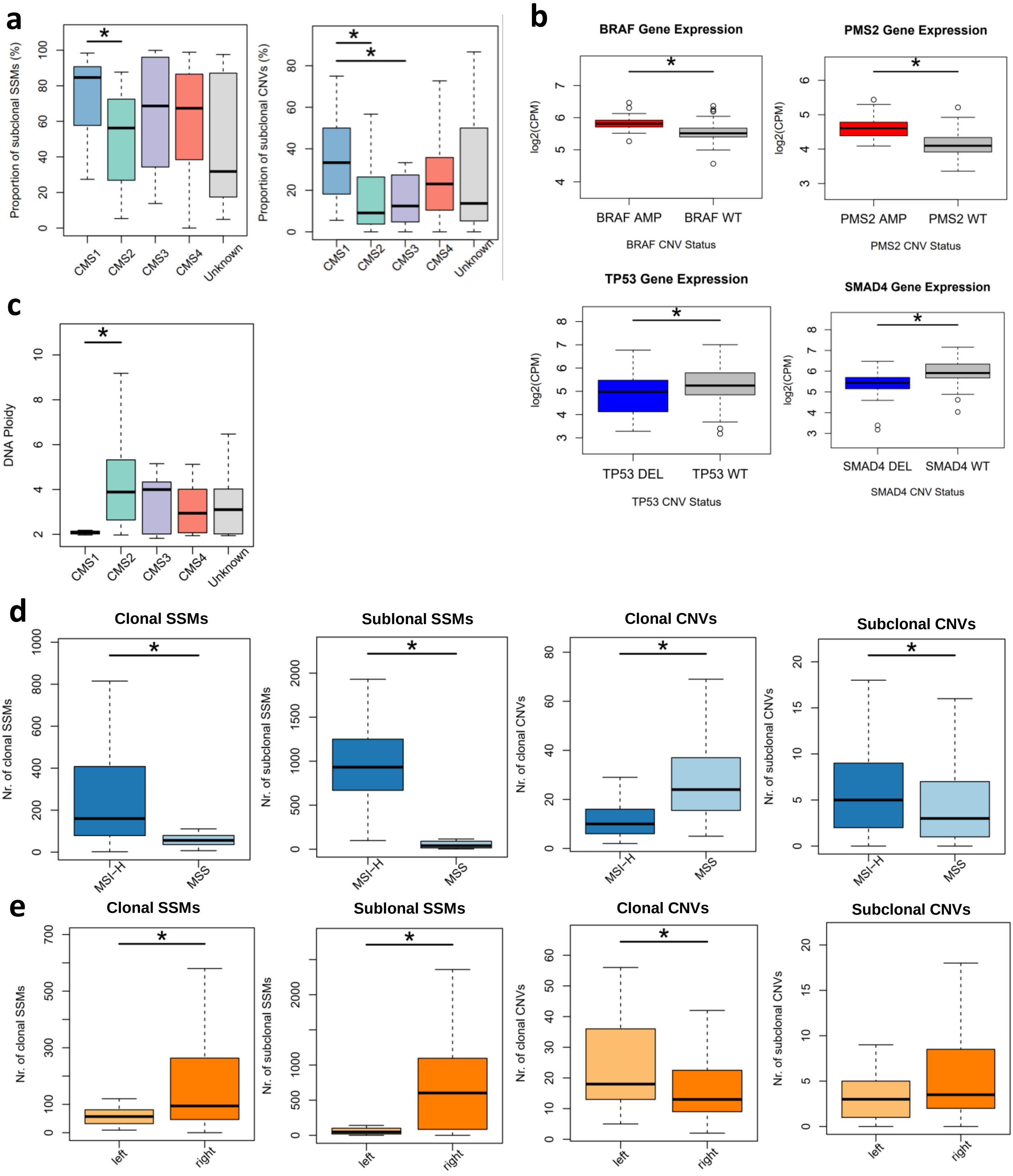

Supplementary Figure 4. Clonal and Subclonal genomic alterations. a) Proportion of subclonal SSMs and CNVs (percentagem relative to total number) for CRC samples segregated according to CMS subtypes. **b)** Expression levels (log CPMs) of genes affected by clonal CNVs in CMS2 subtype. **c)** Distribution of DNA ploidy for CRC samples segregated according to CMS subtypes. **d-e**) Distribution of clonal SSMs, subconal SSMs, clonal CNVs and subclonal CNVs for CRC samples segregated according to MSI status (**d**) and primary tumor location (**e**). *Wilcoxon signed-rank test p-value < 0.05.

Supplementary Figure 5

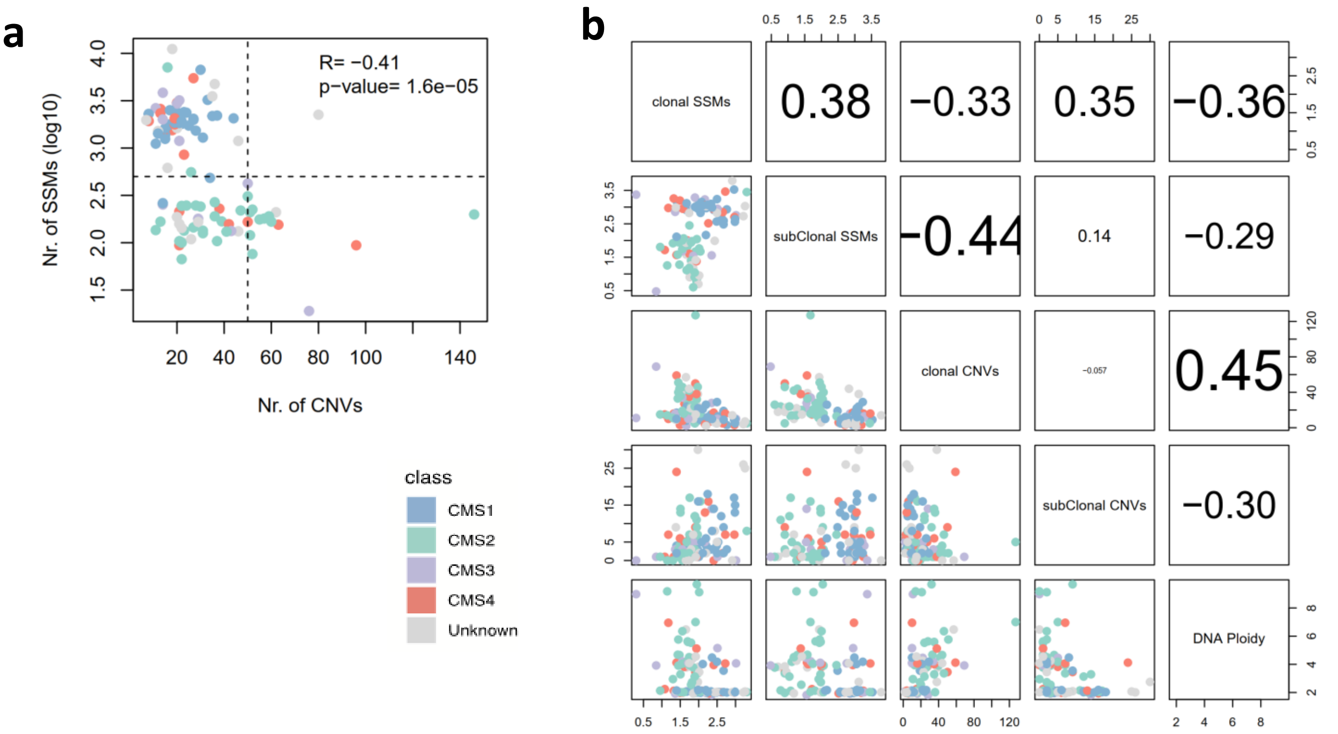

Supplementary Figure 5. SSMs versus CNVs. a-b) Comparison between number of SSMs and CNVs (**a**) and clonal/subclonal SSMs/CNVs and DNA ploidy (**b**). Colors indicate CMS subtype for each CRC sample. SSMs are represented in logarithm (log10). Estimate and statistical significance of the Pearson correlation are presented. *Wilcoxon signed-rank test p-value < 0.05.

Supplementary Figure 6

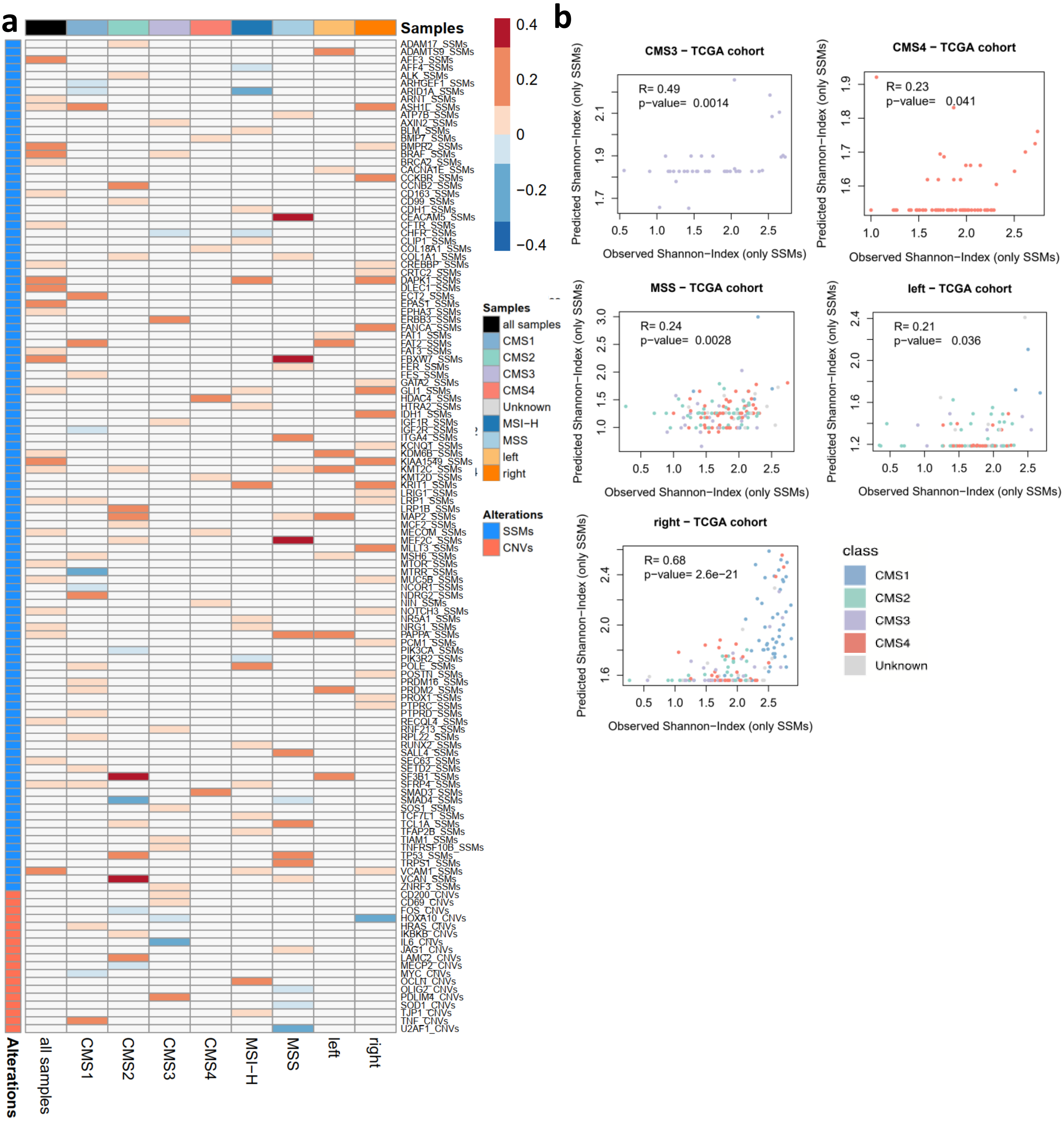

Sup. Figure 6. Genetic biomarker signatures for genetic ITH estimated through Expands (only SSMs). a) Heatmap of cancer-related genes affected by SSMs or CNVs that are associated with genetic ITH levels (only SSMs) in CRC samples depicted by a LASSO penalized model. Each column represents an independent analysis applied to the CRC samples segregated according to CMS subtypes, MSI status or primary tumor location. LASSO-selected coefficients are colored according to the effect of each standardized covariate in the optimal model. Lasso models did not identify any significant gene for Unknown group. **b**) Comparison between observed and predicted genetic ITH levels (only SSMs) for in TCGA cohort for some specific models. Colors indicate CMS subtype for each CRC sample. Estimate and statistical significance of the Pearson correlation are presented. R2 represents the explained variance of the model in our cohort.

Supplementary Figure 7

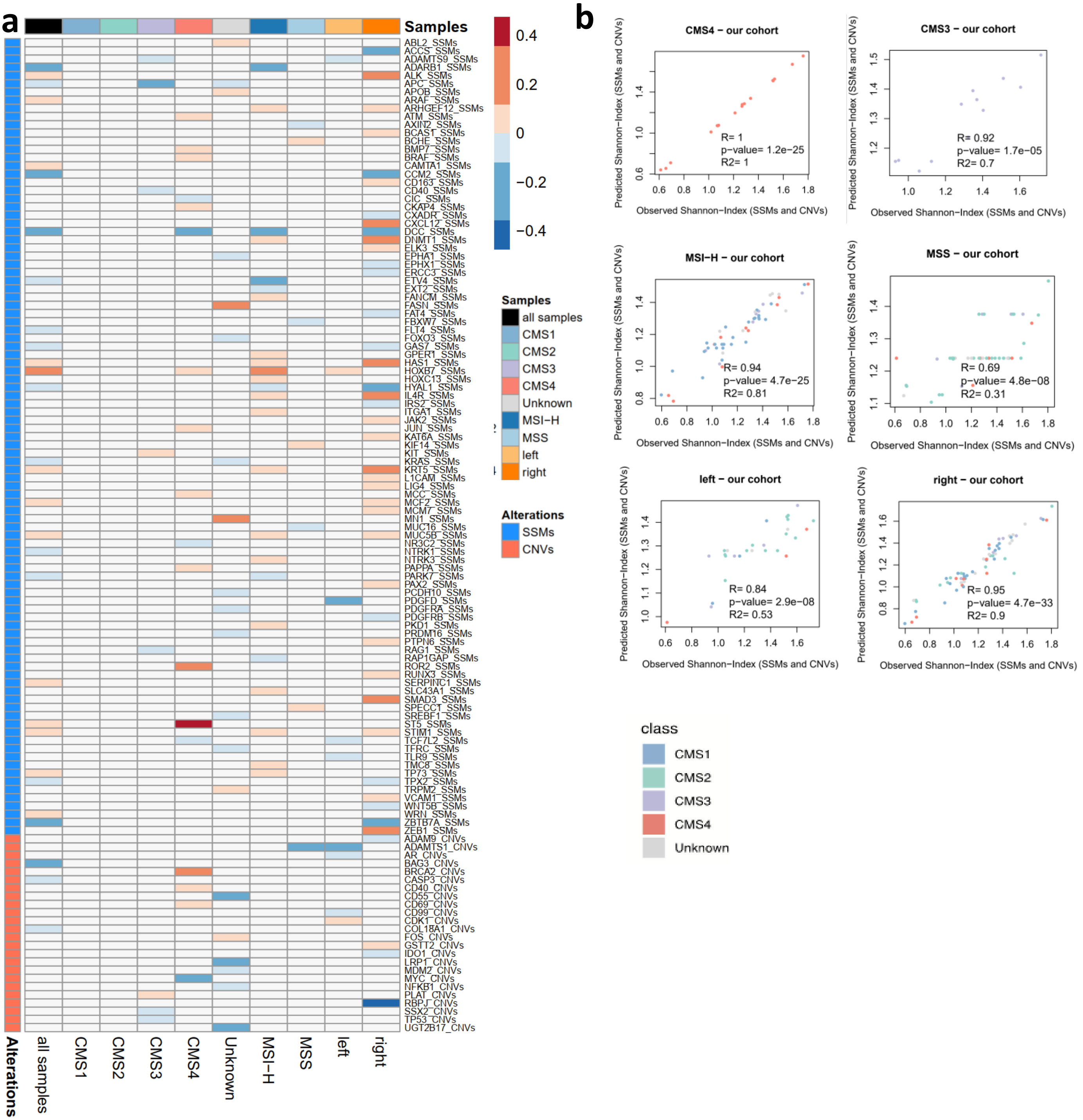

Supplementary Figure 7. Genetic biomarker signatures for genetic ITH estimated through PhyloWGS (based on SSMs and CNVs). a) Heatmap of cancer-related genes affected by SSMs or CNVs that are associated with genetic ITH levels (SSMs and CNVs) in CRC samples depicted by a LASSO penalized model. Each column represents an independent analysis applied to the CRC samples segregated according to CMS subtypes, MSI status or primary tumor location. LASSO-selected coefficients are colored according to the effect of each standardized covariate in the optimal model. Lasso models were not obtained for CMS1 and CMS2 groups. **b**) Comparison between observed and predicted genetic ITH levels (only SSMs) for our cohort for some specific models. Colors indicate CMS subtype for each CRC sample. Estimate and statistical significance of the Pearson correlation are presented. R2 represents the explained variance of the model in our cohort.

Supplementary Figure 8

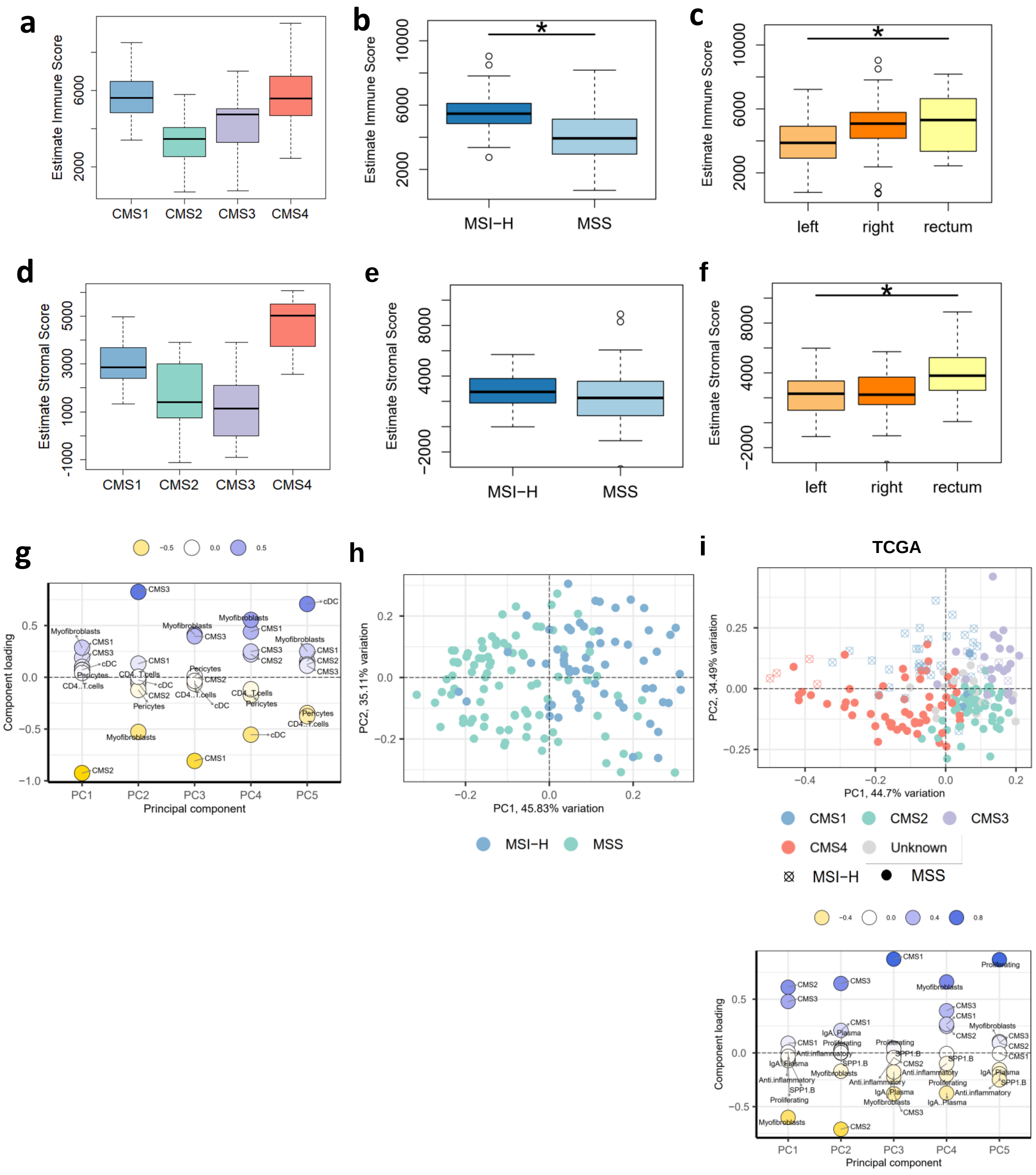

Supplementary Figure 8. Tumor Microenvironment of CRC. a-e) Distribution of imune and stromal scores for CRC samples sgregated according to CMS groups; MSI status and primary tumor location. **g**) Loadings of Principal Component Analysis of Figure 3a. **h**) Principal Component Analysis of Figure 3a coloring samples according to MSI status. **i**) Principal Component Analysis of cell frequencies from RNA-based deconvolution approach for TCGA cohort, and respective loadings. CRC samples are colored according to CMS subtype.

Supplementary Figure 9

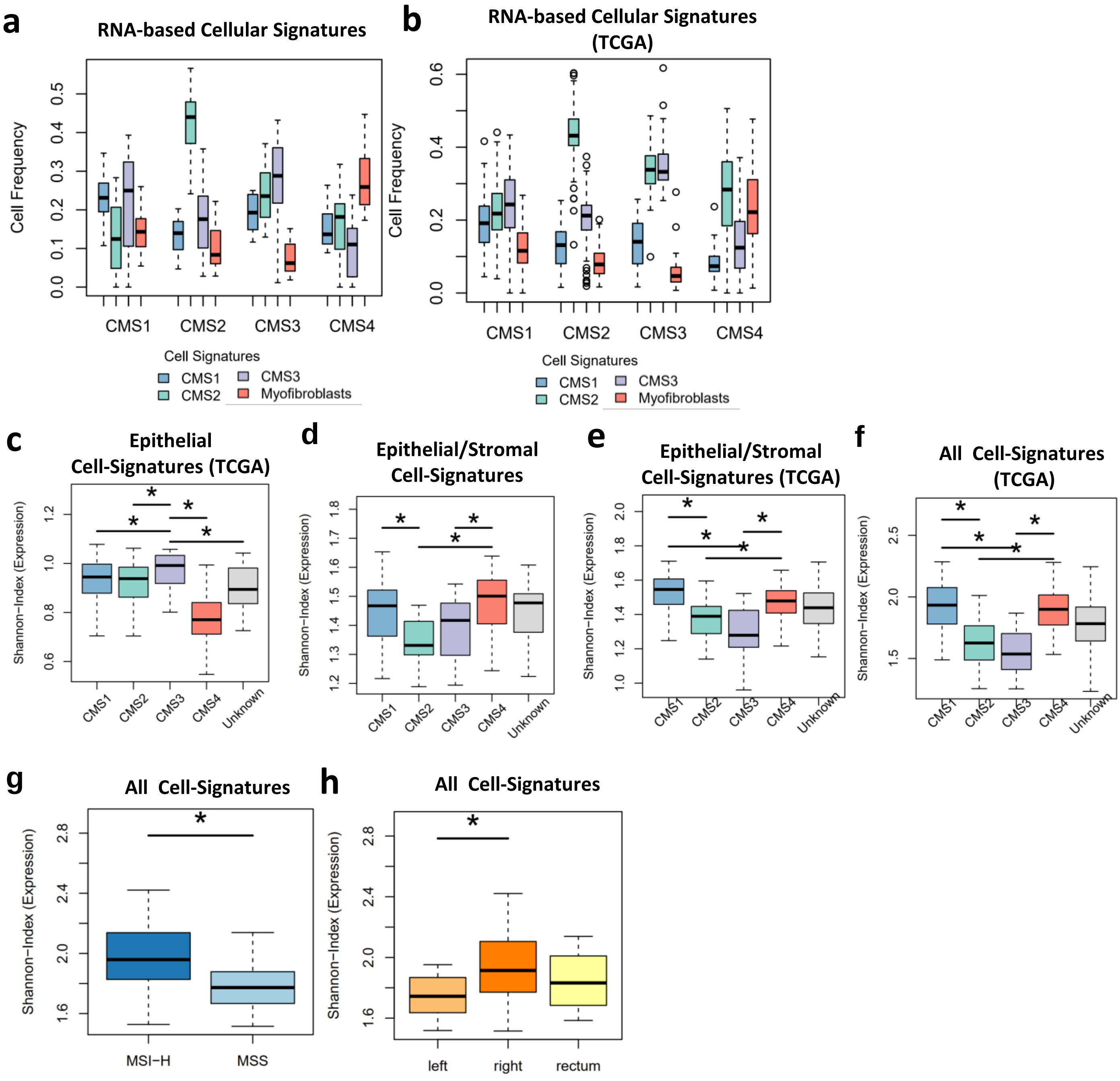

Supplementary Figure 9. Microenvironment Inter- and Intra-Tumor Heterogeneity of CRC. a-b) Frequency of the cell type markers for each CMS subtype, as determined by Cibersort for our cohort (**a**) and TCGA cohort (**b**). **c-f**) Distribution of the microenvironment Shannon-Index (based on expression signatures) of CRC samples segregated by CMS subtype, considering only epithelial cells for TCGA cohort (**c**); only epithelial and stromal cells in our cohort (**d**) and TCGA cohort (**e**); all cell-signatures in TCGA cohort (**f**). **g-h**) Distribution of the microenvironment Shannon-Index (based on expression signatures) using all cell-signatures for CRC samples segregated by MSI status (**g**) and primary tumor location (**h**). *Wilcoxon signed-rank test p-value < 0.05.

Supplementary Figure 10

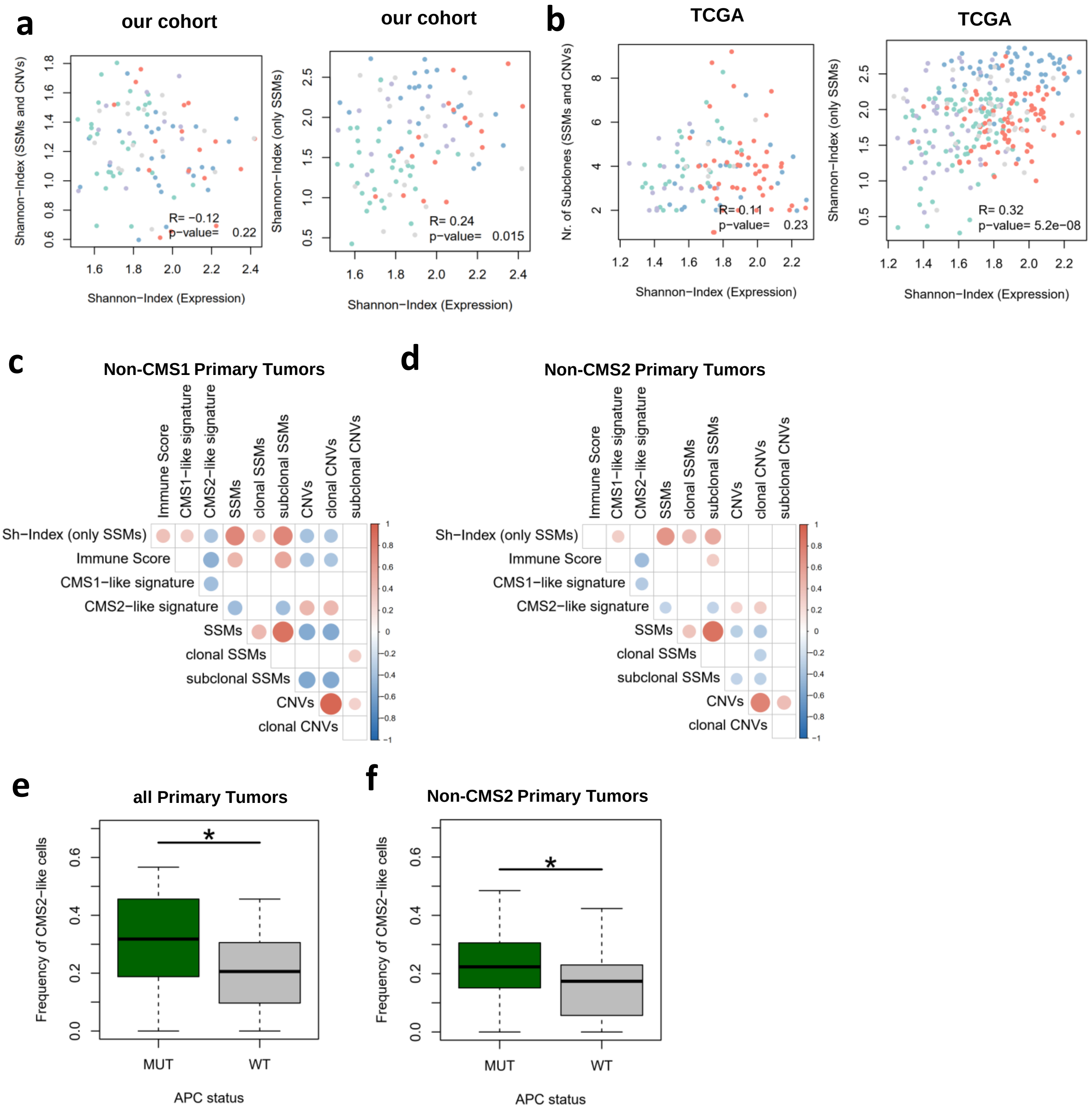

Supplementary Figure 10. Genetic versus Microenvironment Heterogeneity. a-b) Comparison between genetic and microenvironment heterogeneity for our cohort (**a**) and TCGA cohort (**b**). Colors indicate CMS subtype for each CRC sample. Estimate and statistical significance of the Pearson correlation are presented. R2 represents the explained variance of the model in our cohort. **c-d**) Heatmap of Spearman correlation coefficient between: genetic Shannon-Index (only SSMs); immune score from Estimate; cell frequencies of CMS1/CMS2-like signatures; and number of SSMs and CNVs (total, clonal and subclonal) for non-CMS1 (**c**) and non-CMS2 tumors (**d**). **e-f**) Distribution of CMS2-like cells in all tumor samples (**e**) and non-CMS2 tumors (**f**). *Wilcoxon signed-rank test p-value < 0.05.

Supplementary Figure 11

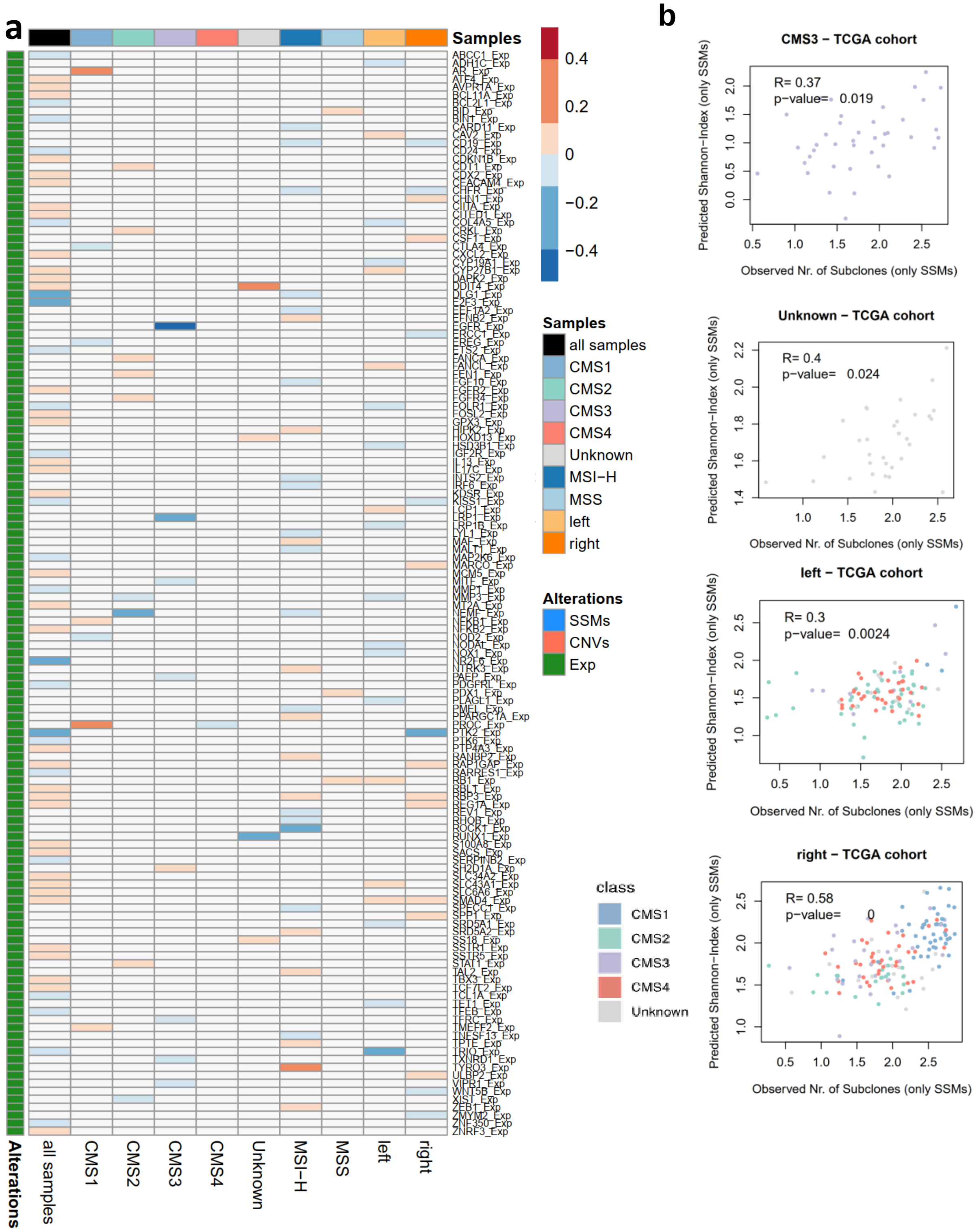

Supplementary Figure 11. Transcriptional biomarker signatures for genetic ITH estimated through Expands (only SSMs). a) Heatmap of cancer-related genes whose expression levels are associated with genetic ITH levels (only SSMs) in CRC samples depicted by a LASSO penalized model. Each column represents an independent analysis applied to the CRC samples segregated according to CMS subtypes, MSI status or primary tumor location. LASSO-selected coefficients are colored according to the effect of each standardized covariate in the optimal model. **b**) Comparison between observed and predicted genetic ITH levels (only SSMs) for in TCGA cohort for some specific models. Colors indicate CMS subtype for each CRC sample. Estimate and statistical significance of the Pearson correlation are presented. R2 represents the explained variance of the model in our cohort.

Supplementary Figure 12

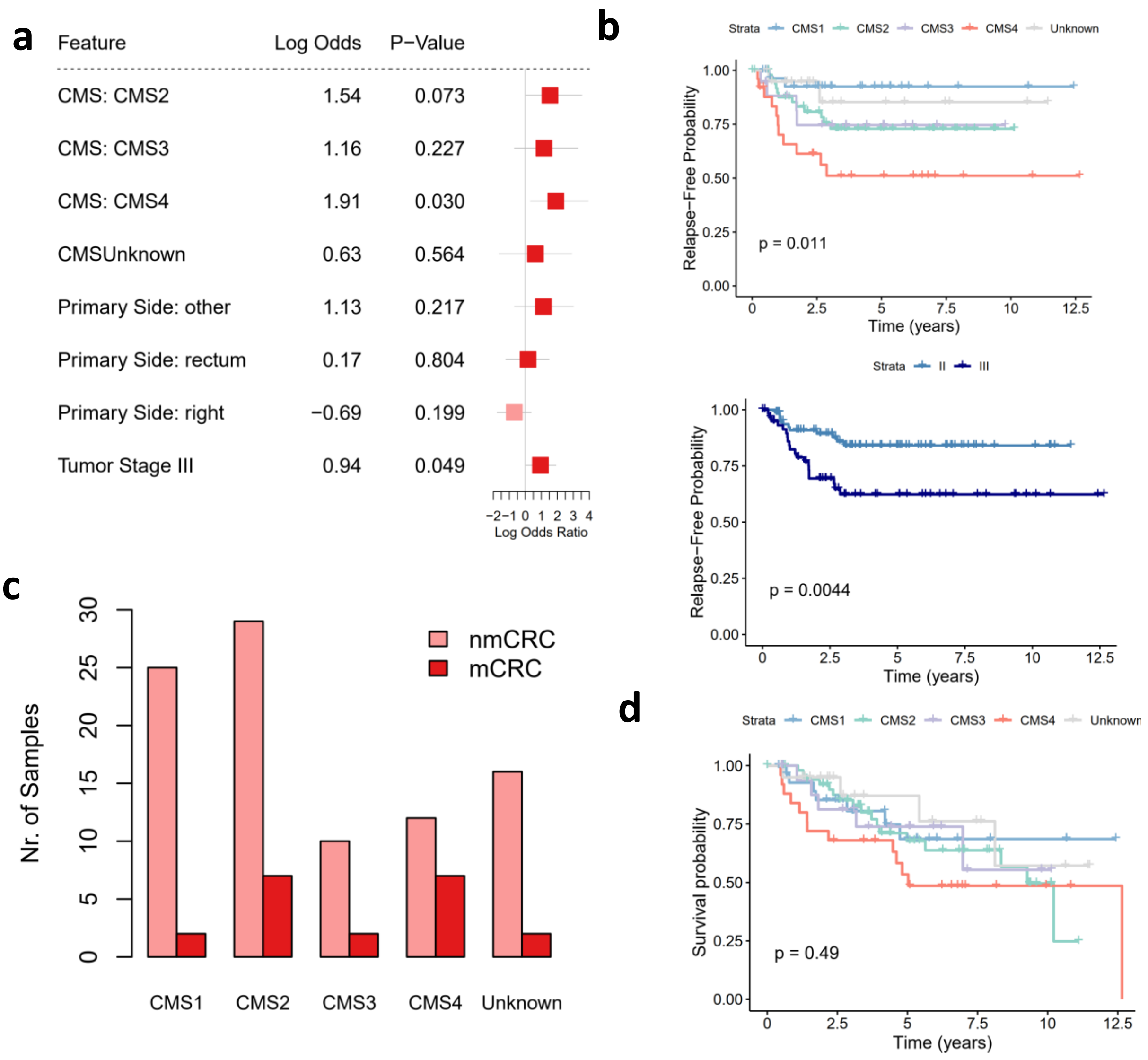

Supplementary Figure 12. Clinical features and Metastatic Potential. a) Coefficients (log-odds ratios) of generalized linear models for metastatic potential considering: CMS classification, primary tumor location and stage. **b**) Kaplan-Meier plot comparing the relapse-free curve of CRC patients segregated according to CMS group and Tumor Stage; **c**) Number of samples that relapsed for each CMS group. **d**) Kaplan-Meier plot comparing the survival curve of CRC patients segregated according to CMS group. Log-rank test p-value is represented.

Supplementary Figure 13

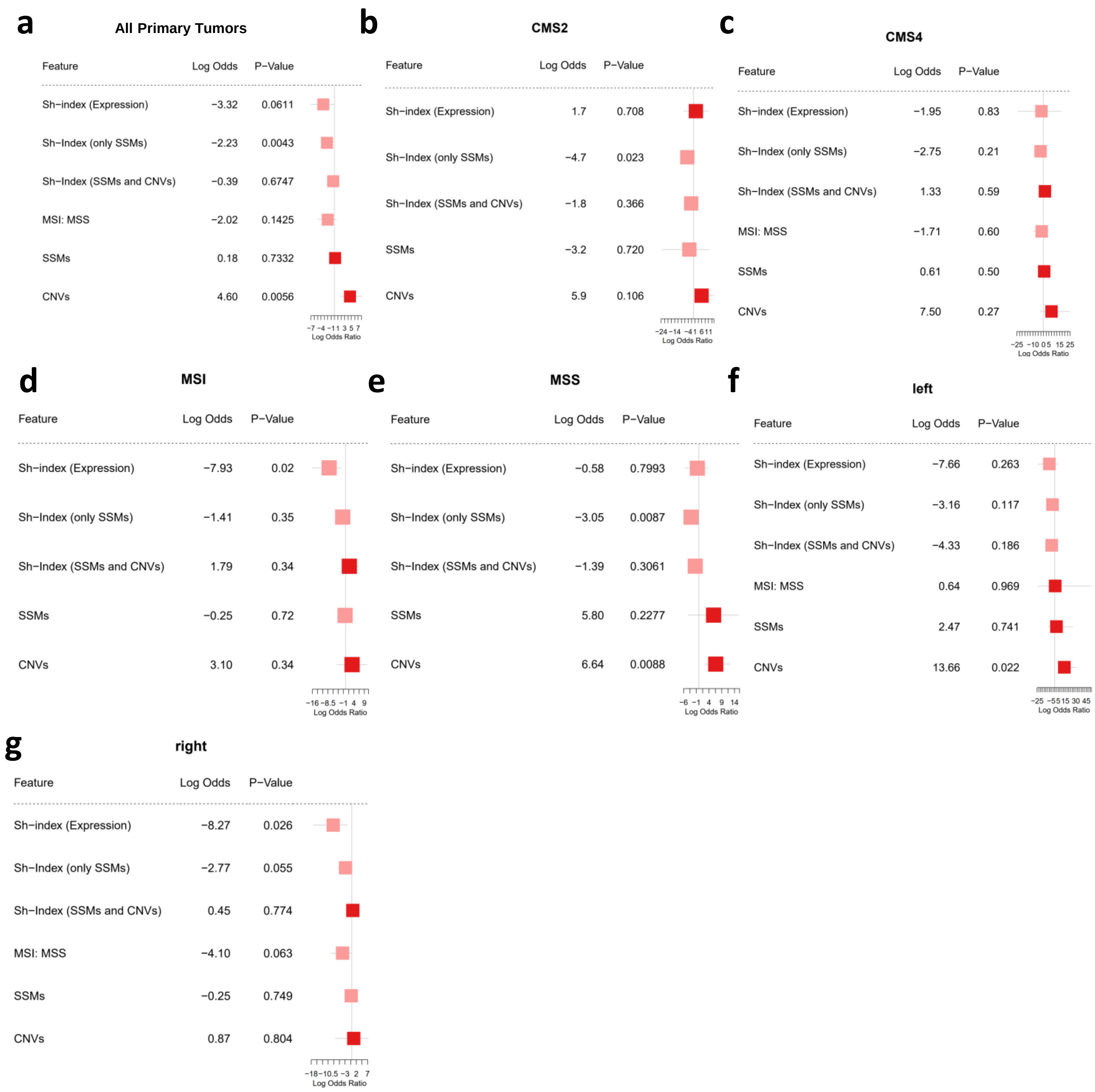

Supplementary Figure 13. Tumor Heterogeneity and Metastatic Potential. a-g) Coefficients (log-odds ratios) and statistical significance of generalized linear models for metastatic potential considering intra-tumor heterogeneity (genetic and microenvironment); number of genomic alterations (SSMs and CNVs).

Supplementary Figure 14

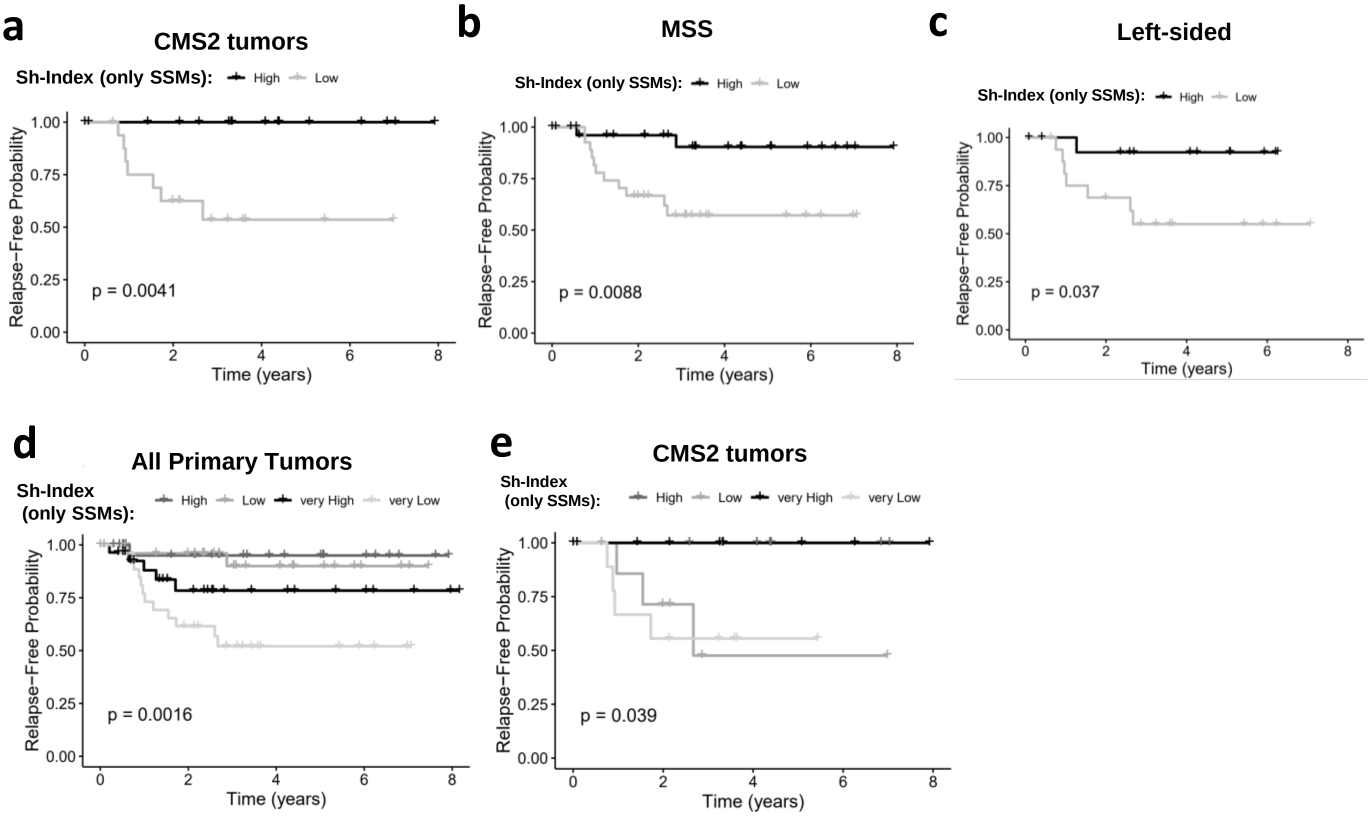

Supplementary Figure 14. Tumor Heterogeneity and Metastatic Potential. a-c) Kaplan-Meier plot comparing the relapse-free curve of CRC patients segregated according to genetic intra-tumor heterogeneity levels (only SSMs), considering two groups (low - gray; high - black) in CMS2 subtype (**a**), MSS (**b**) and left-sided tumors (**c**); **d-e**) Kaplan-Meier plot comparing the relapse-free curve of CRC patients segregated according to genetic intra-tumor heterogeneity levels (only SSMs), considering four groups (very low - light gray; low - gray; high - dark gray; very high - black) for all primary tumors (**d**) and CMS2 tumors (**e**). Log-rank test p-value is represented.

Supplementary Figure 15

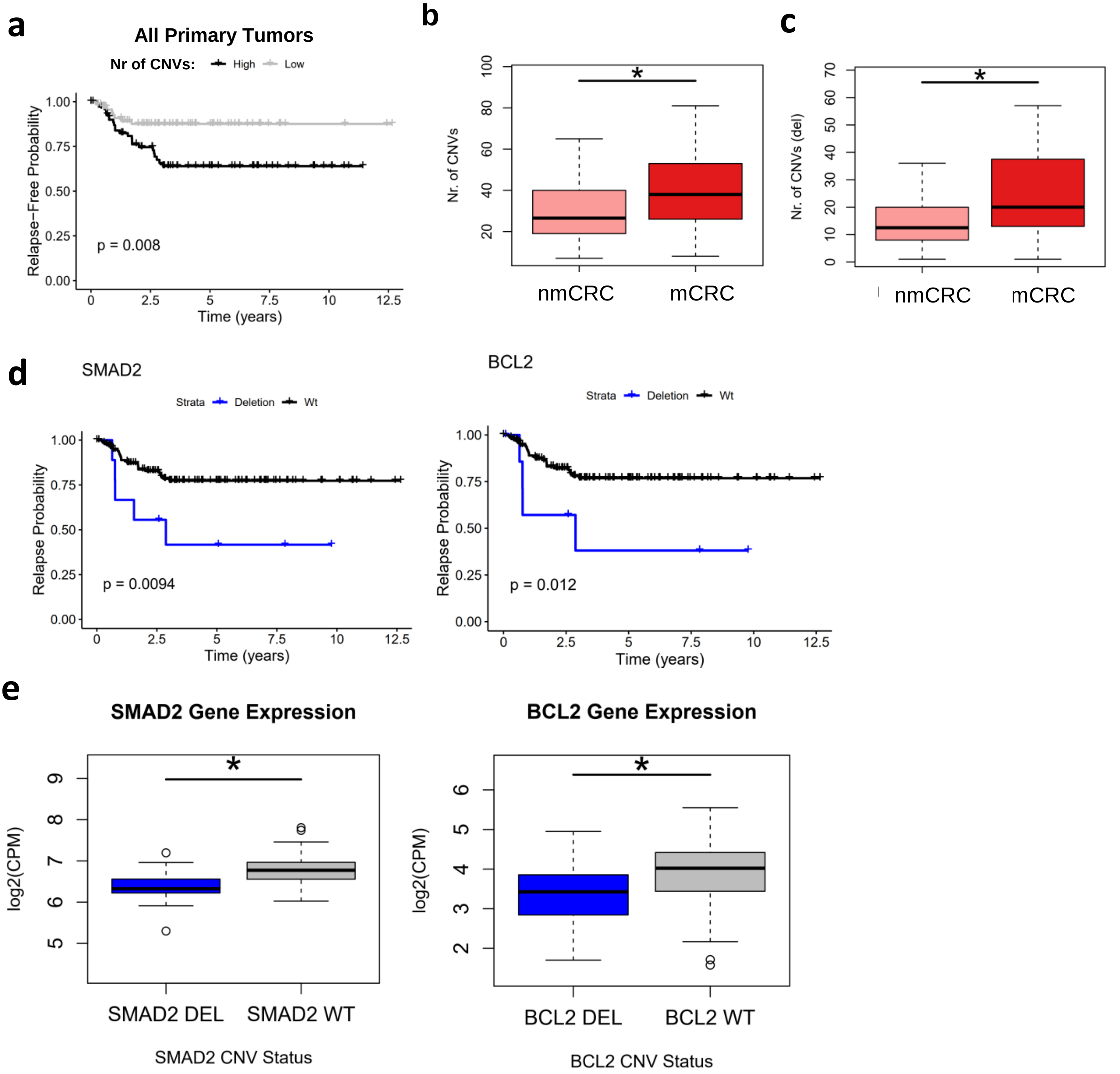

Supplementary Figure 15. CNVs and Metastatic Potential. a) Kaplan-Meier plot comparing the relapse-free curve of CRC patients segregated according to the number of CNVs: low (gray); and high (black). Log-rank test p-value is represented. **b-c)** Number of CNVs (**b**) and deletions (**c**) in CRC samples segregated according to metastasis relapse. **d**) Kaplan-Meier plot comparing the relapse-free curve of CRC patients segregated according to presence of deletions in *SMAD2* and *BCL2* genes. Log-rank test p-value is represented. **e**) Expression levels (log CPMs) of genes affected by deletions.

Supplementary Figure 16

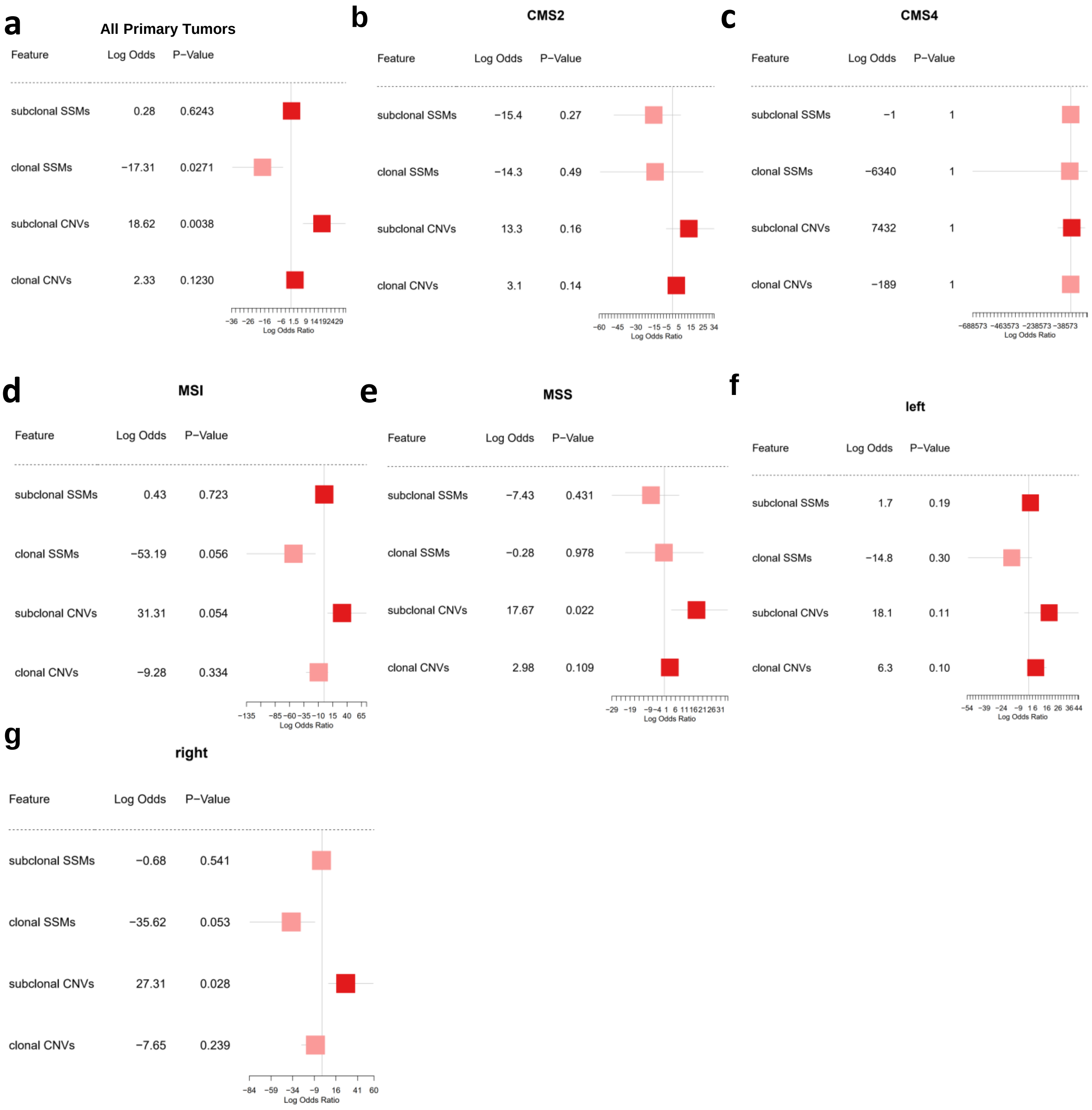

Supplementary Figure 16. Subclona/clonal alterations and Metastatic Potential. a-g) Coefficients (log-odds ratios) and statistical significance of generalized linear models for metastatic potential clonal and subclonal genomic alterations (SSMs and CNVs).

Supplementary Figure 17

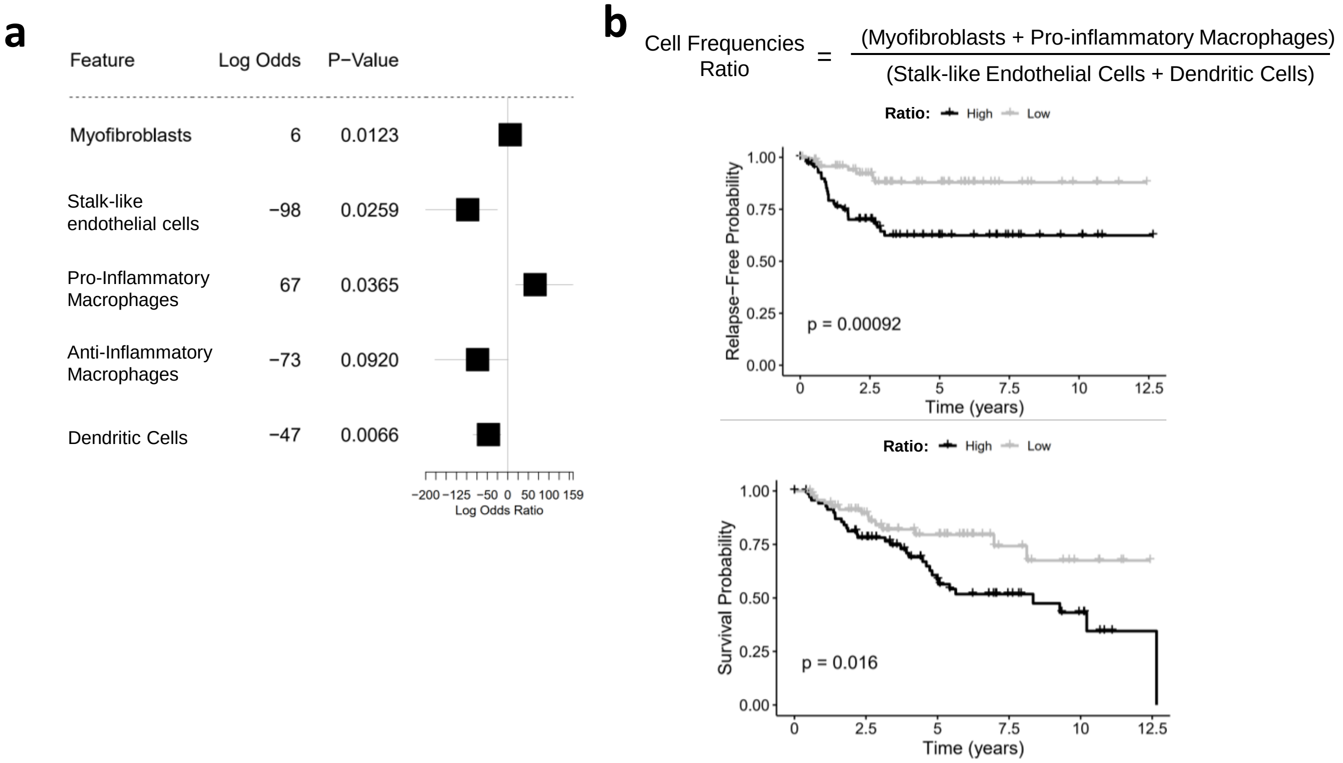

Supplementary Figure 17. Microenvironment Heterogeneity and Metastatic Potential. a) Coefficients (log-odds ratios) and statistical significance of generalized linear models for metastatic potential considering cell frequencies of RNA-based cell signatures. **b**) Kaplan-Meier plot comparing the relapse-free and survival curves of CRC patients segregated according to ratio between pro and anti-metastatic cell frequencies. Log-rank test p-value is represented.

Supplementary Figure 18

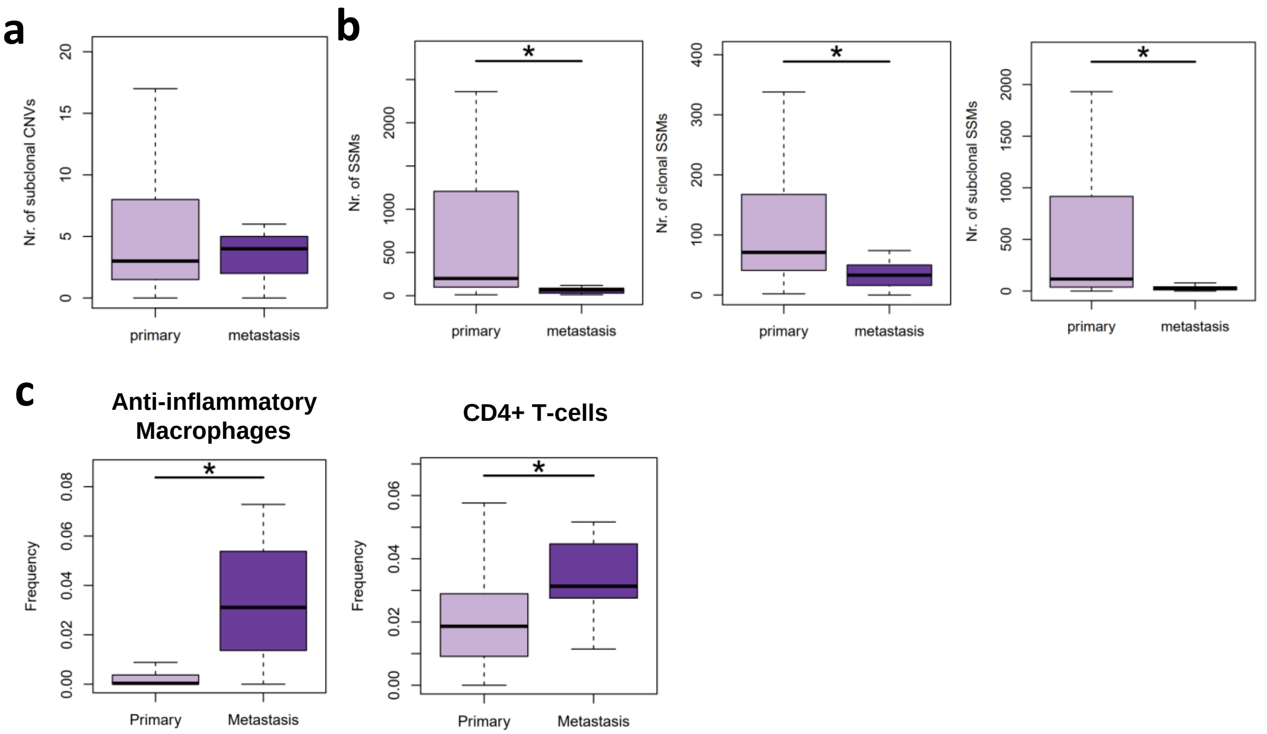

Supplementary Figure 18. Clonal diversity and Metastasis Development. a) Number of subclonal CNVs in primary tumors and metastases. **b**) Number of total, clonal and subclonal SSMs in primary tumors and metastasis. **c)** RNA-based cell frequencies for anti-inflammaotory and CD4+ T-cells in primary tumors and metastasis. *Wilcoxon signed-rank test p-value < 0.05.

Supplementary Figure 19

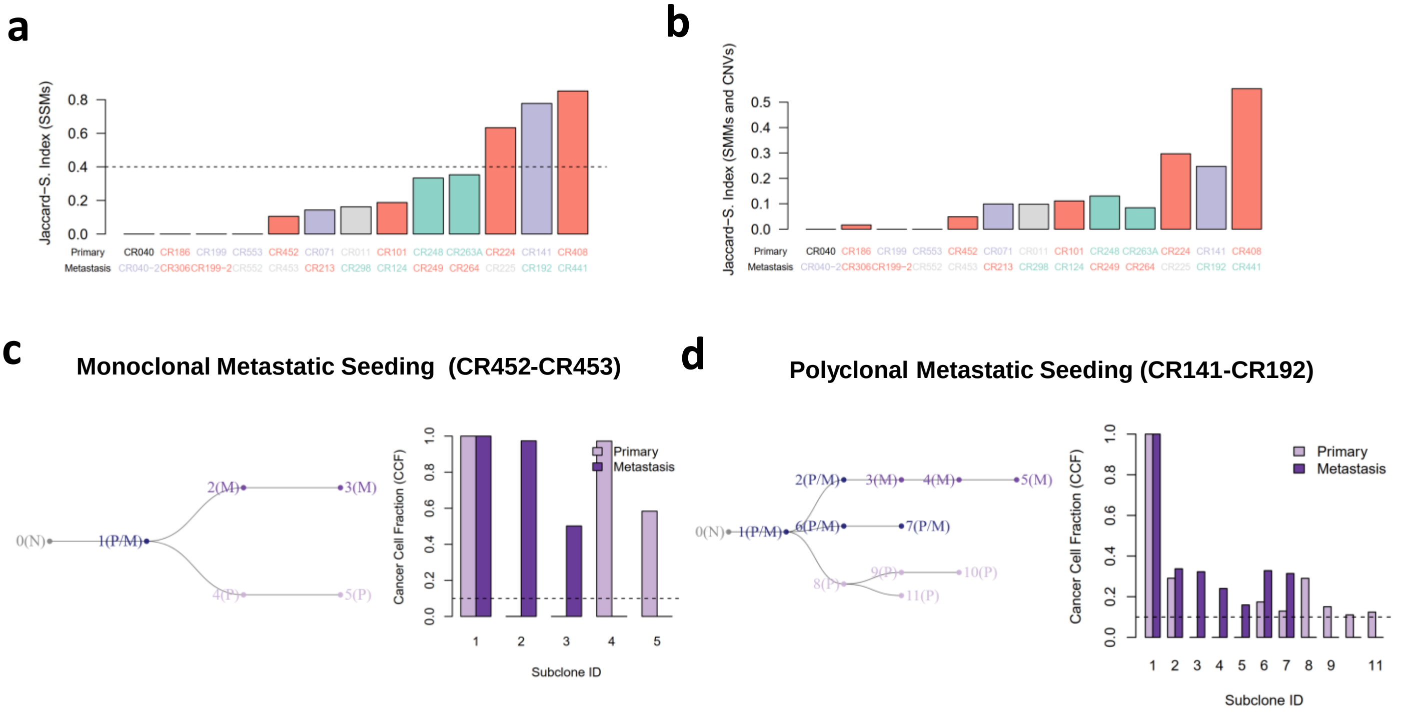

Supplementary Figure 19. Clonal diversity and Metastasis Development in primary-metastasis pairs of our cohort. a-b) Jaccard Similatiry Index (JSI) for each primary-metastasis pair based only on SSMs (**a**) or combining SSMs and CNVs (**b**). **c-d**) Phylogenetic trees depicting subclonal expansion for primary-metastasis pairs with monoclonal (**c**) and polyclonal metastic seeding (**d**). The subclones are identified with the respective number and the containing sample: normal (N), primary tumor (P) and metastasis (M). The barplots represent the cancer cell fraction of each subclones in primary and metastasis.

Supplementary Figure 20

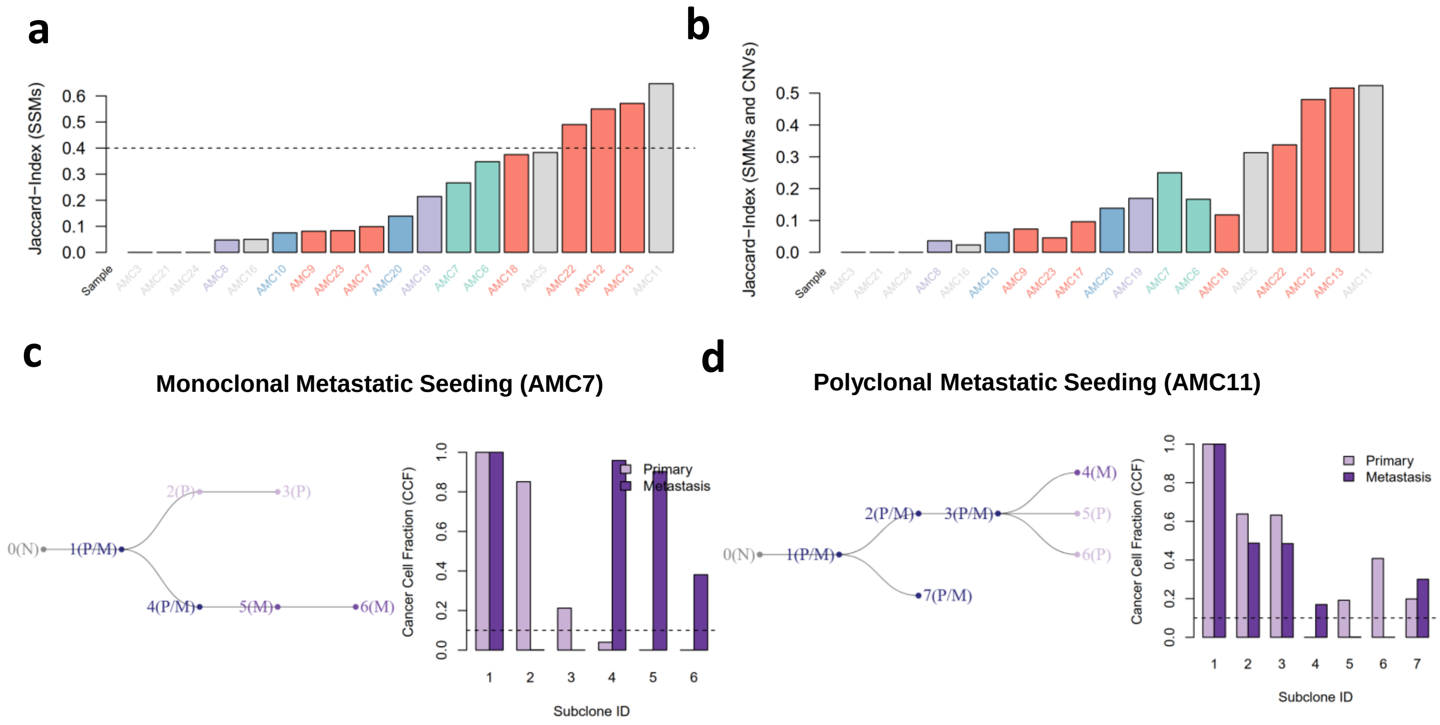

Supplementary Figure 20. Clonal diversity and Metastasis Development in primary-metastasis pairs of a public cohort (Li et al 2015). a-b) Jaccard Similatiry Index (JSI) for each primary-metastasis pair based only on SSMs (**a**) or combining SSMs and CNVs (**b**). **c-d**) Phylogenetic trees depicting subclonal expansion for primary-metastasis pairs with monoclonal (**c**) and polyclonal metastic seeding (**d**). The subclones are identified with the respective number and the containing sample: normal (N), primary tumor (P) and metastasis (M). The barplots represent the cancer cell fraction of each subclones in primary and metastasis.

Supplementary Figure 21

a

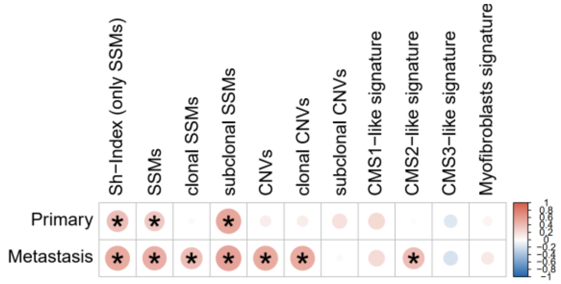

b

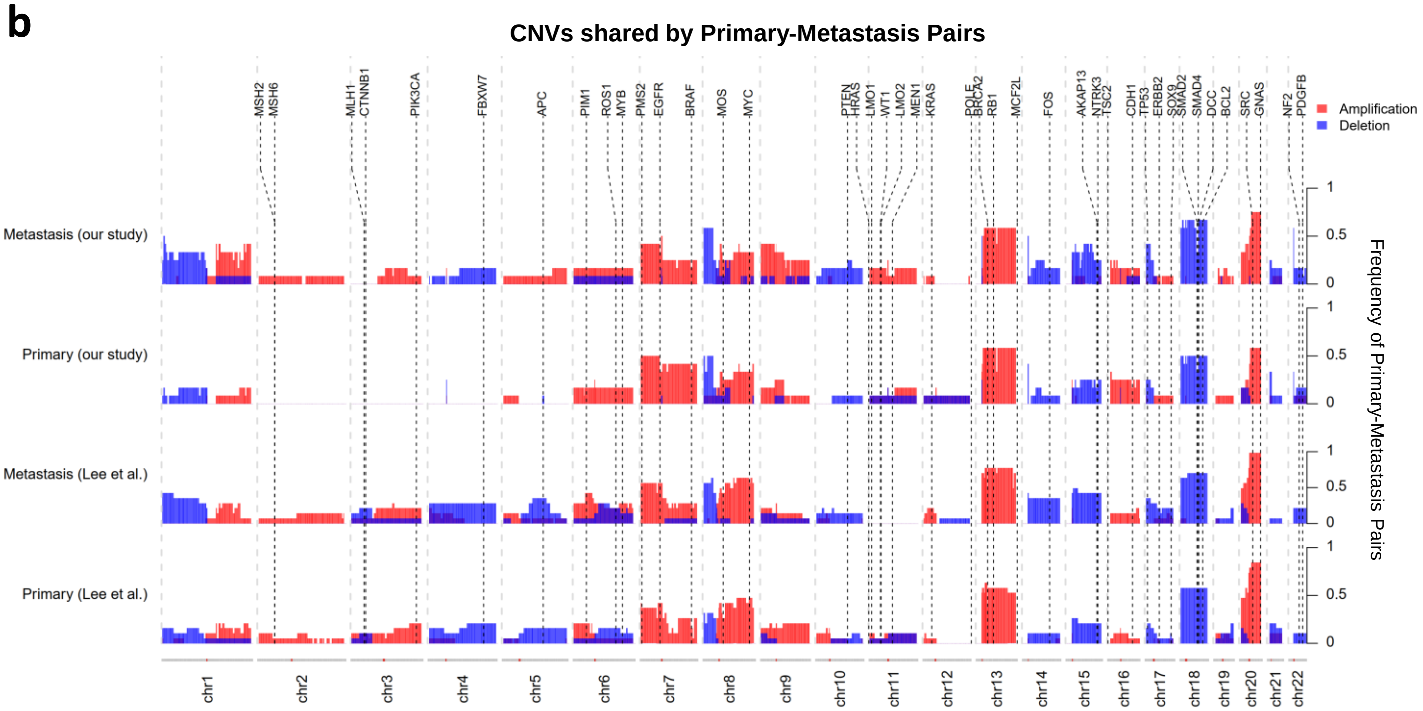

Supplementary Figure 21. CNVs and tumor evolution. a) Heatmap of correlations between Jaccard-Similarity Index and several molecular features for paired primary and metastases: genetic Shannon-Index (only SSMs); number of SSMs and CNVs (total, clonal and subclonal); cell frequencies of CMS-like and Myofibroblasts signatures. *Significant correlations (adj. p-value < 0.05). **b**) Frequencies of copy number events (separated in amplifications and deletions) affecting different regions of the genome, in the paired primary-metastasis samples from our study (n=12) and the study of Lim et al. (n=19). Positions of known cancer-related genes are also displayed.
