# Supplementary material for: Genetic and microenvironmental intra-tumor heterogeneity impacts colorectal cancer evolution and metastatic development
Source: Commun Biol. 2022 Sep 9;5:937. doi: 10.1038/s42003-022-03884-x (PMC9463147; doi:10.1038/s42003-022-03884-x)
Supplement: Supplementary file 3 — Description of Additional Supplementary Files [file 42003_2022_3884_MOESM3_ESM.pdf]

Description of Additional Supplementary Files

File name: Supplementary Data
Description: Source Data used to generate the main figures.
